# Mitochondria, endothelial cell function, and vascular diseases

**DOI:** 10.3389/fphys.2014.00175

**Published:** 2014-05-06

**Authors:** Xiaoqiang Tang, Yu-Xuan Luo, Hou-Zao Chen, De-Pei Liu

**Affiliations:** State Key Laboratory of Medical Molecular Biology, Department of Biochemistry and Molecular Biology, Institute of Basic Medical Sciences, Chinese Academy of Medical Sciences and Peking Union Medical CollegeBeijing, China

**Keywords:** mitochondria, endothelial cell, atherosclerosis, diabetes mellitus, pulmonary artery hypertension, hypertension, antioxidants, caloric restriction

## Abstract

Mitochondria are perhaps the most sophisticated and dynamic responsive sensing systems in eukaryotic cells. The role of mitochondria goes beyond their capacity to create molecular fuel and includes the generation of reactive oxygen species, the regulation of calcium, and the activation of cell death. In endothelial cells, mitochondria have a profound impact on cellular function under both healthy and diseased conditions. In this review, we summarize the basic functions of mitochondria in endothelial cells and discuss the roles of mitochondria in endothelial dysfunction and vascular diseases, including atherosclerosis, diabetic vascular dysfunction, pulmonary artery hypertension, and hypertension. Finally, the potential therapeutic strategies to improve mitochondrial function in endothelial cells and vascular diseases are also discussed, with a focus on mitochondrial-targeted antioxidants and calorie restriction.

## Introduction

Mitochondria are the remnants of aerobic bacteria that invaded protoeukaryotic cells a billion years ago. Mitochondria are cell's metabolic headquarters, fueling oxidative phosphorylation for adenosine 5'-triphosphate (ATP) production, and driving reactions to manufacture core metabolites for the biosynthesis of fats, nucleotides, and proteins. The roles of mitochondria in eukaryotic cells go beyond their capacity to act as a metabolic mediator. These cellular organelles regulate various cellular processes, including proliferation (Mitra et al., 2009), immune response (Zhou et al., 2011a), apoptotic cell death (Kroemer et al., 2007), and mediates secondary massager signals to the nucleus (Al-Mehdi et al., 2012).

The ability to utilize oxygen drives the development and evolution of the cardiovascular system in multicellular organisms (Dromparis and Michelakis, 2013). In normal vascular systems, mitochondria regulate various processes in addition to providing ATP for the vascular cells. In diseased human vascular tissues, mitochondria change morphologically and functionally. Mice lacking mitochondrial proteins typically die at the exact time in development when the cardiovascular system forms, or are more sensitive to risk factors for cardiovascular system (Miller et al., 2010; Shenouda et al., 2011; Dong et al., 2013; Kröller-Schön et al., 2013). The functions of mitochondria in individual types of vascular cell have attracted increasing scientific attention.

The normal artery contains three layers. The inner layer, the tunica intima, is lined by a monolayer of endothelial cells (EC) that is in contact with blood. The middle layer, or tunica media, contains smooth muscle cells embedded in a complex extracellular matrix. The adventitia, the outer layer of artery, contains mast cells, nerve endings and microvessels (Libby et al., 2011). The direct contact of ECs with the blood flow means that they are particularly vulnerable to damage molecules in the blood on one hand, and that they have ideally “guard” roles on the other hand (i.e., sensing alterations in perfusate constituents and either responding directly or transmitting reactive signals to nearby cells, such as smooth vascular cells) (Davidson and Duchen, 2007). Endothelial dysfunction contributes to the development of nearly all vascular diseases. Even though ECs have low mitochondria content, mitochondrial dynamics acts as a pivotal orchestrator of EC homeostasis under normal conditions, damage of mitochondrial dynamics participates in endothelial dysfunction and diverse vascular diseases. In this review, we summarize advances in understanding the roles of mitochondria in ECs and the mechanisms by which EC mitochondria participate in certain vascular diseases, including atherosclerosis, diabetic endothelial dysfunction, pulmonary artery hypertension (PAH) and hypertension. Finally, we discuss briefly current available mitochondria targeting approaches for the treatment of vascular diseases.

## Mitochondria in endothelial function

### Mitochondrial content, subcellular localization, biogenesis and dynamics in endothelial cells

In comparison with other cell types with higher energy requirements, mitochondria content in ECs is modest. In rat ECs, for example, mitochondria compose 2–6% of the cell volume as opposed to 28% in hepatocytes and 32% in cardiac myocytes (Dromparis and Michelakis, 2013; Kluge et al., 2013). The low content of mitochondria in ECs may indicate that mitochondria-dependent oxidative phosphorylation is not that important for energy supplement in those cells. In fact, ECs obtain a large proportion of their energy from the anaerobic glycolytic metabolism of glucose. In cultured pig aortic ECs, more than 75% of ATP is provided by glycolysis (Culic et al., 1997). Additionally, 99% of glucose is catabolized into lactate in isolated coronary microvascular ECs, whose oxygen consumption is mainly attributable to the oxidation of endogenous substrates (Spahr et al., 1989; Mertens et al., 1990). Actually, mitochondria are more likely to serve primarily as essential signaling organelles in the vascular endothelium (Quintero et al., 2006).

The cellular distribution of mitochondria is important for its function and its communication with other cellular organelle (especially endoplasmic reticulum, ER) and nucleus. For example, in ECs of arterioles isolated from human myocardium, mitochondria are anchored to the cytoskeleton. Those mitochondria release ROS in response to cell deformation by shear stress (Liu et al., 2008). In addition, exposure of pulmonary artery ECs to hypoxia triggers a retrograde mitochondrial movement that requires microtubules and the microtubule motor protein dynein, resulting in the perinuclear clustering of mitochondria. This subcellular redistribution of mitochondria is accompanied by the accumulation of ROS in the nucleus, which can be attenuated by suppressing perinuclear clustering of mitochondria with nocodazole to destabilize microtubules (Al-Mehdi et al., 2012). In addition, mitochondria are an important Ca^2+^ buffering system that cooperates with ER to maintain cellular Ca^2+^ hemostasis. In this regard, the sublocation and interaction with ER are critical for the role of mitochondria in Ca^2+^ buffering (Mironov et al., 2005). Indeed, distance between the ER and mitochondria is associated with intramitochondrial calcium, mitochondrial membrane potential (Δψ_m_) and mitochondria-dependent apoptosis in artery vascular cells from PAH (Sutendra et al., 2011).

The content of mitochondria, which is important for cellular function, is critically regulated in cells. Cellular mitochondria content depends upon the balance between mitochondrial biogenesis and mitophagy. The biogenesis of mitochondria is a complex and incompletely understood process involving replication of mitochondria DNA (mtDNA) and expression of nuclear and mitochondrial genes. New mitochondria formation is regulated by the peroxisome proliferation-activated receptor γ co-activator 1α (PGC-1α), which activates nuclear respiratory factor (NRF)-1, NRF-2, transcription factor A mitochondrial (TFAM), and transcription factor B mitochondrial (TFBM). Mitochondria are dynamic organelles that constantly fuse and divide (collectively termed mitochondrial dynamics) and can build large, interconnected intracellular networks. During the lifespan of mitochondria, damage accumulates followed by mitochondrial fusion and fission to generate functionally normal and damaged mitochondria. Healthy mitochondria re-enter the cycles of fusion and function in cells whereas the damaged ones depolarize and undergo mitophagy (Kluge et al., 2013). These concerted activities control mitochondrial morphology and intracellular distribution and determine their cell type-specific appearance. The antagonistic and balanced activities of the fusion and fission machineries shape the mitochondrial compartment, and the dynamic behavior of mitochondria allows cells to respond to their physiological conditions. A shift toward fusion favors the generation of interconnected mitochondria, whereas a shift toward fission produces numerous mitochondrial fragments (Westermann, 2010). In recent years, research on mitochondrial fusion and fission has gained much more attention, as it is important for our understanding of many biological processes, including the maintenance of mitochondrial functions, apoptosis and aging, in ECs (Detmer and Chan, 2007). Defects in mitochondria biogenesis and dynamics have detrimental consequences on bioenergetic supply and contribute to the endothelial dysfunction and the pathogenesis of cardiovascular diseases (Ong et al., 2010; Shenouda et al., 2011). In addition, the observation that mitochondria biogenesis is inhibited in aging vascular ECs is important, particularly because systemic vascular diseases such as atherosclerosis and hypertension are often diseases of the aging population (Dromparis and Michelakis, 2013).

### Mitochondrial ROS in endothelial cells

**Figure 1:**
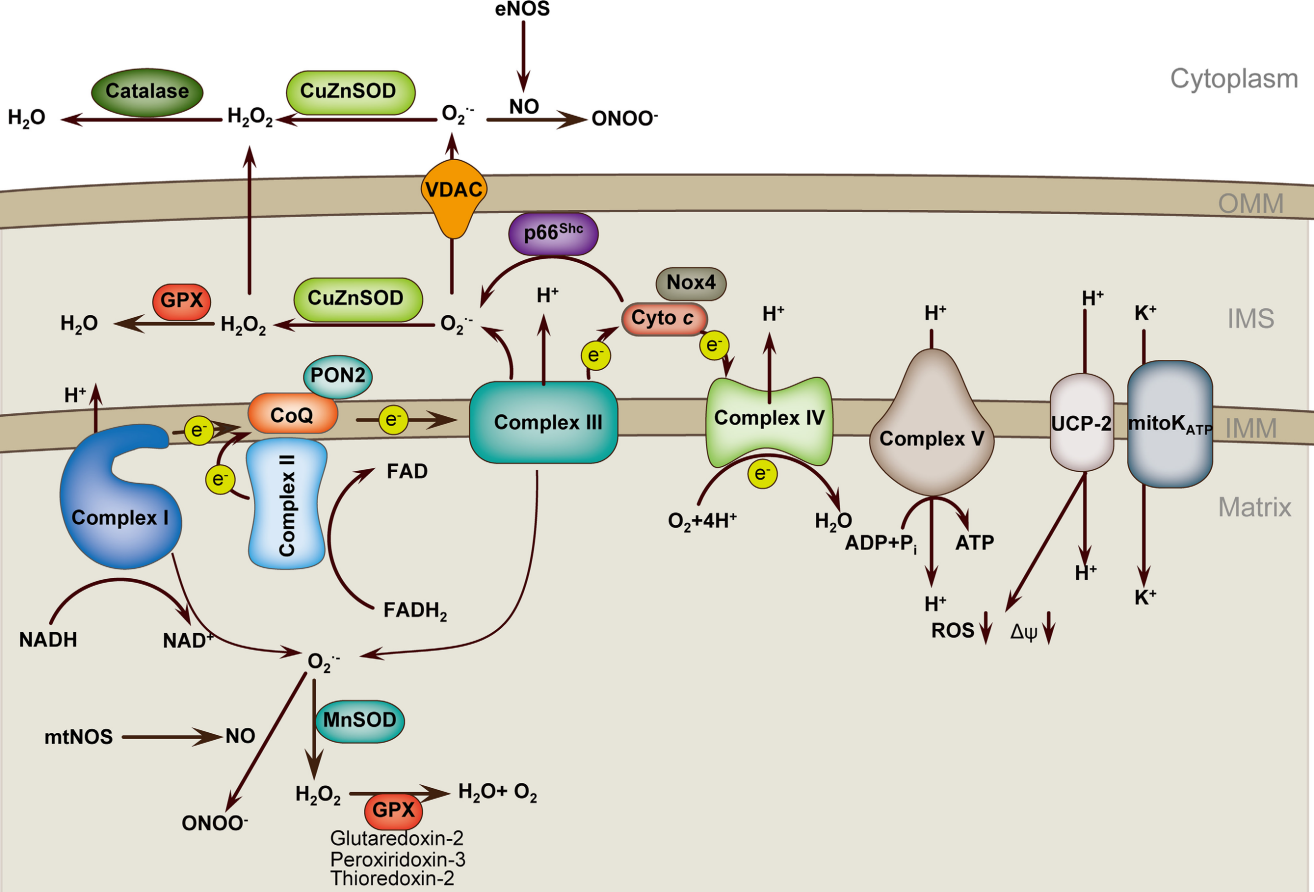
**Mitochondria ROS regulation in endothelial cell**. Respiratory chain complexes I–IV generate the proton gradient over the mitochondrial inner membrane that drives ATP generation by ATP synthase (complex V). Electrons (e^−^) from NADH and FADH2 pass through complex I and complex II, respectively, and then to complex III *via* the co-enzyme ubiquinol (CoQ). Cytochrome *c* transfers electrons from complex III to complex IV, which reduces O_2_ to form H_2_O. Flow of electrons is accompanied by proton (H^+^) transfer across the inner mitochondrial membrane (IMM) at complexes I, III, and IV, creating an electrochemical gradient, Δψ_m_. Protons reenter the mitochondrial matrix through complex V, which uses the proton-motive force to generate ATP. UCPs and mitoK_ATP_ allow protons to return to the matrix, reducing ROS formation. Complex I leaks electrons to generate O^▪^_2_ toward the matrix, whereas complex III generates O^▪^_2_ toward both matrix and intermembrane space (IMS). p66^Shc^ in the IMS subtracts electrons from cytochrome *c* to produce O^▪^_2_. Superoxide is dismutated to H_2_O_2_ by CuZnSOD in IMS and by MnSOD in the matrix. H_2_O_2_ is reduced to H_2_O by glutathione peroxidase (GPX) using GSH, and the resultant oxidized glutathione (GSSG) is reduced back to GSH by glutathione reductase. O^▪^_2_ can interact with NO to form ONOO^▪^, which may cooperate with O^▪^_2_ to uncoupling eNOS and amplify ROS production. PON2, Paraoxonase 2; NOX4, nicotinamide adenine dinucleotide phosphate oxidase 4; UCP2, uncoupling protein 2; mitoK_ATP_, mitochondrial ATP-sensitive potassium channel; OMM, outer mitochondrial membrane.

Mitochondria are important source of reactive oxygen species (ROS) and serve as important ROS buffering systems (Figure 1). Mitochondria can sense danger signals such as infectious agents or cholesterol crystals. Mitochondria-derived reactive oxygen species (mROS) are critical signals for the initiation of cellular responses to stress and disease risk factors. Altered Δψ_m_ is an important factor that triggers excess mROS production in the setting of risk factors, including aging, hypercholesterolemia, hyperglycemia, smoking, infections and hypoxia. Although most electrons flowing down the electron transport chain (ETC) redox gradient ultimately reach complex V, 1–3% of electrons prematurely react with oxygen, at complexes I and III, to form superoxide and other types of ROS, collectively known as mROS (Dromparis and Michelakis, 2013). In addition to complexes I and III, other sources of mROS have been identified in ECs. One such example is the nicotinamide adenine dinucleotide phosphate (NADPH) oxidase 4 (Nox4), which is highly expressed in ECs and has been localized to mitochondria in other tissues, although mitochondrial localization in ECs remains elusive. Nox4 is the most highly expressed Nox family member in all cells of the cardiovascular system and is upregulated by a wide variety of agonists and cellular stresses. In ECs, Nox4 is sensitive to mechanical forces. Nox4 and its homolog Nox2 are required for basal ROS production and EC proliferation (Lassègue et al., 2012). Unlike Nox1, endogenous Nox4 predominantly produces H_2_O_2_ rather than O^▪−^_2_ (Dikalov et al., 2008). Interestingly, a current report supports that Nox4 is a protective ROS-generating vascular NADPH oxidase partly through preventing endothelial dysfunction during ischemic or inflammatory stress (Schröder et al., 2012). Another example is the growth factor adaptor protein p66^Shc^, which functions in mitochondrial signaling. p66^Shc^ facilitates the generation of H_2_O_2_ by oxidizing cytochrome *c* (Giorgio et al., 2005; Paneni et al., 2012). The next source of mROS to be introduced is the mitochondrial ATP-sensitive potassium channel (mitoK_ATP_). Although the function of mitoK_ATP_ in ECs is not well-investigated, current evidence shows that pharmacological mitoK_ATP_ activation protects against ischemic cell death in cultured ECs and prevents endothelial vasodilator function in Langendorff-perfused guinea pig hearts subjected to ischemia-reperfusion. In addition, inhibition of mitoK_ATP_ channels also represses high-glucose-induced endothelial cell apoptosis (Beresewicz et al., 2004; Feng and Zuo, 2011; Huang et al., 2012).

Once excessive mROS is produced, cells simply and rapidly response to oxidative stress by directly targeting the excessive mROS. Manganese superoxide dismutase (MnSOD), which is the predominant dismutase in mitochondria, is rapidly inducible and buffers the superoxide in the mitochondria matrix by dismutating superoxide to H_2_O_2_ (Dromparis and Michelakis, 2013). Other superoxide dismutases, such as CuZnSOD, buffer the superoxide that escapes into the intermembranous space and cytoplasm or even extracellularly. The levels of H_2_O_2_ are downregulated by antioxidant enzymes, including catalase and peroxidases. Catalase is located in cytosolic peroxisomes. Important mitochondrial peroxidases include thioredoxin-2, peroxididoxin-3, and glutaredoxin-2. Glutathione peroxidase-1 is located both in mitochondria and in the cytoplasm of ECs (Kluge et al., 2013). In addition to superoxide dismutase, other mitochondria proteins may also participate in the buffering of mROS. Paraoxonase 2 (PON2) is one member of the PON gene family that consists of three proteins (PON1, PON2, and PON3). PON2 is an intracellular membrane-associated protein that is widely expressed in vascular cells. PON2 protein is localized to the inner mitochondrial membrane, where it is associated with respiratory complex III. PON2 binds with high affinity to coenzyme Q10, an important component of the ETC and reduces the production of mROS (Devarajan et al., 2011). Our previous review in *Antioxidants and Redox Signaling* has systemically discussed the features and functions of the PON gene family (She et al., 2012). Uncoupling proteins (UCPs), a family of five mitochondria-localized proteins, may be another antioxidant defense. UCPs generally tend to limit mROS production. For instance, UCP1 overexpression in ECs inhibits mROS production (Nishikawa et al., 2000; Cui et al., 2006), and UCP2 overexpression in human aortic ECs blocks fatty acid-induced mROS generation (Lee et al., 2005). UCP2 is the primary isoform in ECs. UCP2 critically modulates Δψ_m_ and mROS production (Duval et al., 2002; Lee et al., 2005). UCP2 preserves endothelial function through increasing nitric oxide (NO) bioavailability secondary to the inhibition of ROS production in the endothelium of obese diabetic mice (Tian et al., 2012). UCP2 upregulation also ameliorates hyperglycemia-induced endothelial dysfunction (Sun et al., 2013a).

At relatively low levels, mROS can be critical signaling molecules that support normal or compensatory function of the cell (Sena and Chandel, 2012). This fact means that mROS may increase even as part of normal signaling in the cell while the mitochondria themselves remain normal. mROS are now known to be biologically important in a variety of physiological systems, including adaptation to hypoxia, regulation of autophagy, immunity, differentiation, and longevity. For instance, cells utilize an acute increase in mROS to stabilize hypoxia-inducible factor (HIF) under hypoxia condition and subsequently restrain ROS production in chronic hypoxia to avoid cellular damage (Sena and Chandel, 2012). However, if mROS production is significantly increased (due to increased oxygen levels and mitochondrial metabolism) and exceeds the buffering capacity of MnSOD, oxidative damage and cellular dysfunction or death ensues. The superoxide anion in the matrix is highly reactive and can damage mtDNA, lipids, and proteins. mROS can also damage the high-iron-sulfur-containing ETC complexes themselves, which may further exacerbate mROS production and set up a vicious cycle that contributes to endothelial dysfunction and vascular diseases. In healthy or the early stage of vascular diseases, the combination of mitochondrial dynamics, mitophagy, and biogenesis may replace damaged mitochondria or their components and maintain normal mitochondrial function. Nevertheless, these quality-control mechanisms may be impaired, which may result in retention of dysfunctional mitochondria that produce excess ROS and facilitate vascular diseases. Therefore, mROS are initially considered toxic molecules. Clinical investigations implicate that many vascular diseases are accompanied with elevated mROS levels. The mechanisms underlying mROS in vascular diseases are multiple and complex. One typical mechanism by which mROS participates in endothelial dysfunction and subsequent vascular diseases is by uncoupling the endothelial NO synthase (eNOS). eNOS facilitates the production of NO, which is an antihypertensive, antithrombotic and anti-atherosclerotic molecule. In human umbilical vein endothelial cells (HUVEC), the mitochondrial arginase II is constitutively expressed, whereas the cytosolic arginase I is barely detectable. Endothelial NO synthesis depends on the activity of arginase II in mitochondria and L-arginine carriers in cell membrane (Topal et al., 2006). O^▪−^_2_ reacts with NO to form ONOO^−^, which together with ROS production leads to mitochondrial dysfunction, as evidenced by increased mROS production, depolarization of Δψ_m_, decreased respiratory control ratio, and reduced low-molecular-weight thiols content. Reaction with O^▪−^_2_ limits NO availability, resulting in eNOS uncoupling. As a consequence of eNOS uncoupling, NO production is reduced and the pre-existing oxidative stress is enhanced, which contribute significantly to endothelial dysfunction and vascular diseases (Li and Förstermann, 2013).

Noticeably, endothelial mitochondrial oxidative stress can affect the faith of other cell populations, such as the podocytes in the kidney (Daehn et al., 2014). Endothelial dysfunction promotes podocyte apoptosis. Inhibition of endothelin-1 receptor type A (EDNRA) or scavenging of mROS prevents podocyte loss, albuminuria, glomerulosclerosis, and renal failure (Daehn et al., 2014). The mechanism underlying podocyte apoptosis after endothelial dysfunction may also include the decrease in NO bioavailability, given that loss of eNOS from glomerular EC has been recently demonstrated to affect podocyte function via activatin of RhoA in diabetes (Yuen et al., 2012). In addition, in an eNOS-deficient model, glomerular endothelial cell injury precedes podocyte apoptosis after adriamycin treatment (Sun et al., 2013c).

### Mitochondria and calcium homeostasis in the endothelium

Like other cells, the functions of ECs largely depend on various extents on changes in intracellular Ca^2+^ concentration. For instance, receptor-dependent agonists, such as acetylcholine and serotonin, activate eNOS by increasing cytosolic calcium and stimulating the binding of calcium/calmodulin (Kluge et al., 2013). Briefly, calcium activates calcium/calmodulin-dependent protein kinase II, which plays a role in eNOS gene expression and phosphorylation state, and regulates actin cytoskeletal elements that influence EC shape, motility, and barrier function as previously discussed (Cai et al., 2008).

**Figure 2:**
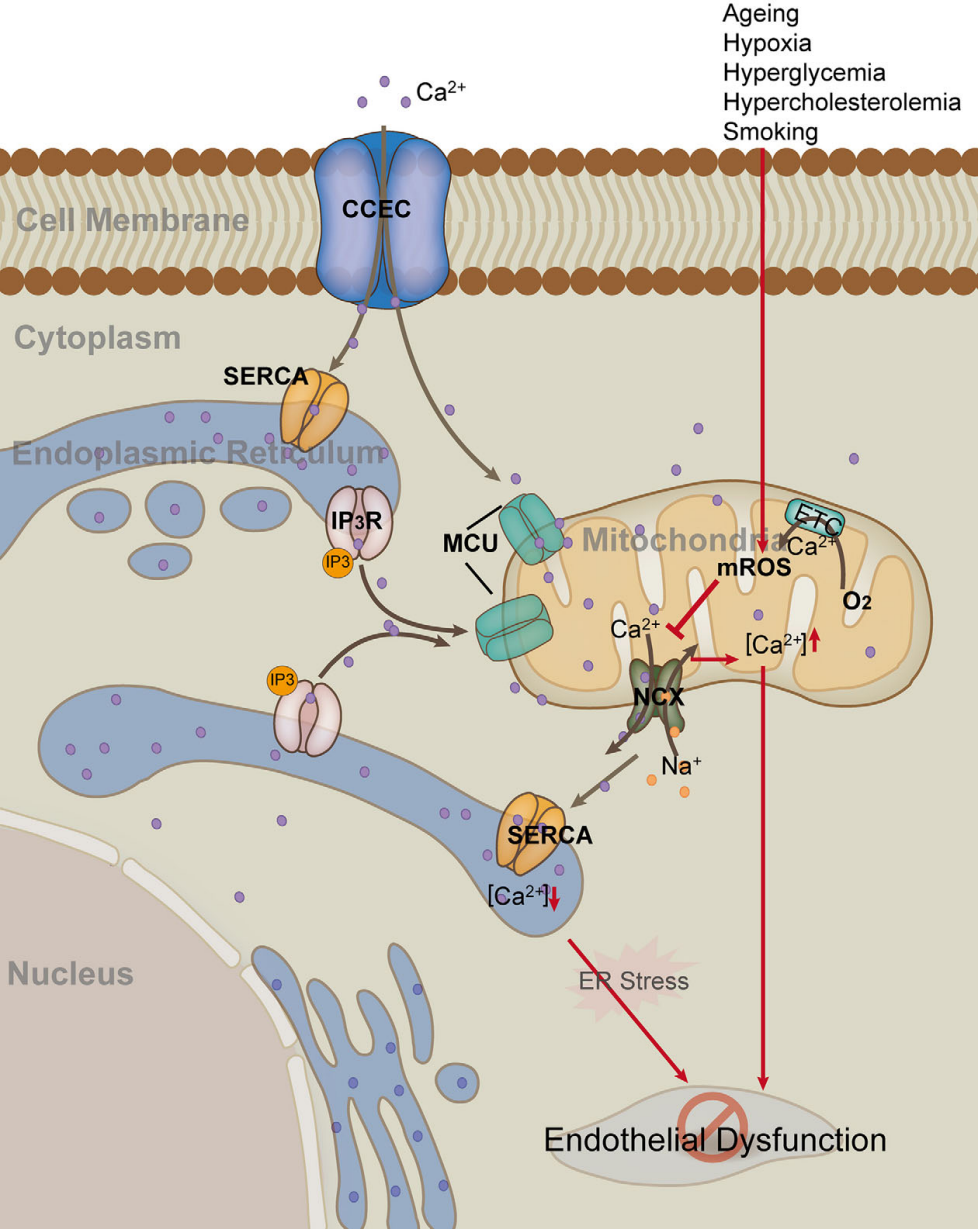
**Mitochondria in endothelial cell calcium hemostasis**. Endothelial cells uptake Ca^2+^ through the membrane capacitative Ca^2+^ entry channel (CCEC). Generally, endoplasmic reticulum (ER) and mitochondria uptake Ca^2+^ through sarco/ER Ca^2+^-ATPase (SERCA) and mitochondrial Ca^2+^ uniporter (MCU), respectively. Inositol 1,4,5-triphosphate (IP3) can activate the release of Ca^2+^ from ER by binding to IP3 receptor on the membrane of ER. In contrast, mitochondria release Ca^2+^ and at the same time uptake Na^+^ through the Na^+^/Ca^2+^ exchanger (NCX). Risk factors such as hypoxia and hyperglycemia, up-regulate mROS production, which in turn inhibits the activity of mitochondrial NCX, leading to elevated level of [Ca^2+^]_m_ and decreased level of [Ca^2+^]_e_. Persistent high level of [Ca^2+^]_m_ and low level of [Ca^2+^]_e_ result in mitochondria damage and ER stress, as well as subsequent endothelial dysfunction (Red arrow).

Although ER is the major storage site for calcium, 25% of cellular calcium is located to mitochondria. Therefore, mitochondria are also considered to be an important calcium buffering system. Mitochondria modulate Ca^2+^ signals by taking up, buffering, and releasing Ca^2+^ at key locations near Ca^2+^ release or influx channels (Figure 2). The mitochondria and ER networks are in very close proximity; actually, the two organelles communicate and cooperate to regulate calcium trafficking and thereby orchestrate key aspects of endothelial function (Kluge et al., 2013). Calcium moving in and out of mitochondria is highly regulated. In ECs, H_2_O_2_-induced increase in mitochondria calcium may depend partly on the decrease of calcium extrusion via inhibiting the sodium/calcium exchanger (NCX) (Jornot et al., 1999). UCP2 and UCP3 are fundamental for mitochondrial Ca^2+^ uniporter (MCU) activity in human ECs (Trenker et al., 2007). Mitochondrial calcium uniporter regulator 1 (MCUR1) is an integral membrane protein required for MCU-dependent mitochondrial Ca^2+^ uptake. MCUR1 binds to MCU and regulates ruthenium-red-sensitive MCU-dependent Ca^2+^ uptake (Mallilankaraman et al., 2012). MICU1 faces the intermembrane space to sense cytoplasmic Ca^2+^ and regulates the Ca^2+^ threshold and cooperactivity of mitochondrial uniporter (Csordás et al., 2013). Mitochondrial Ca^2+^ uptake controls intracellular Ca^2+^ signaling, cell metabolism, cell survival and other cell-type specific functions by buffering cytosolic Ca^2+^ levels and regulating mitochondrial effectors (Rizzuto et al., 2012). The very negative Δψ_m_ allows mitochondria to sequester positive ions such as Ca^2+^ from the cytoplasm. Mitochondrial calcium is an important orchestrator of mitochondrial biogenesis *per se* and increases expression of PGC1-α (Szabadkai and Duchen, 2008). Physiological changes in mitochondrial ([Ca^2+^]_m_) and cytosolic ([Ca^2+^]_c_) calcium concentrations have important regulatory effects on many aspects of mitochondrial functions, including mROS production, energetics, motility, dynamics, and biogenesis (Davidson and Duchen, 2007; Widlansky and Gutterman, 2011; Kluge et al., 2013).

There is increasing evidence that altered mitochondrial calcium contributes to endothelial response to pathological stimuli. For example, mitochondrial calcium uptake stimulates NO production in mitochondria of bovine vascular ECs (Dedkova et al., 2004). Elevated global endothelial concentration of Ca^2+^ promotes activation of eNOS, which, in turn, leads to the generation of NO (Katakam et al., 2013). Pharmacological depolarization of endothelial mitochondria promotes the activation of eNOS by dual pathway involving elevated [Ca^2+^] as well as by phosphoinositide-3 kinase (PI3K)-induced eNOS phosphorylation. Depolarization of mitochondria in ECs promotes cerebral artery vasodilation (Katakam et al., 2013). Furthermore, activation of tumor necrosis factor receptor 1 (TNFR1) ectodomain shedding by mitochondrial Ca^2+^ determines the severity of inflammation in mouse lung microvessels. This compensatory effect blunts the extent of endothelial activation under proinflammatory conditions (Rowlands et al., 2011). Additionally, the functions of many key mitochondrial enzymes, including PDH, are very Ca^2+^-dependent (Dromparis and Michelakis, 2013; Kluge et al., 2013). Calcium activates tricarboxylic acid cycle enzymes and oxidative phosphorylation, thereby increasing ATP production. In addition to its direct effect on metabolic enzyme activity, a decrease in Ca^2+^ influx hyperpolarizes mitochondria, which leads to mitochondrial and endothelial dysfunction.

### Mitochondrial regulation of endothelial senescence, apoptosis, and mitophagy

**Figure 3:**
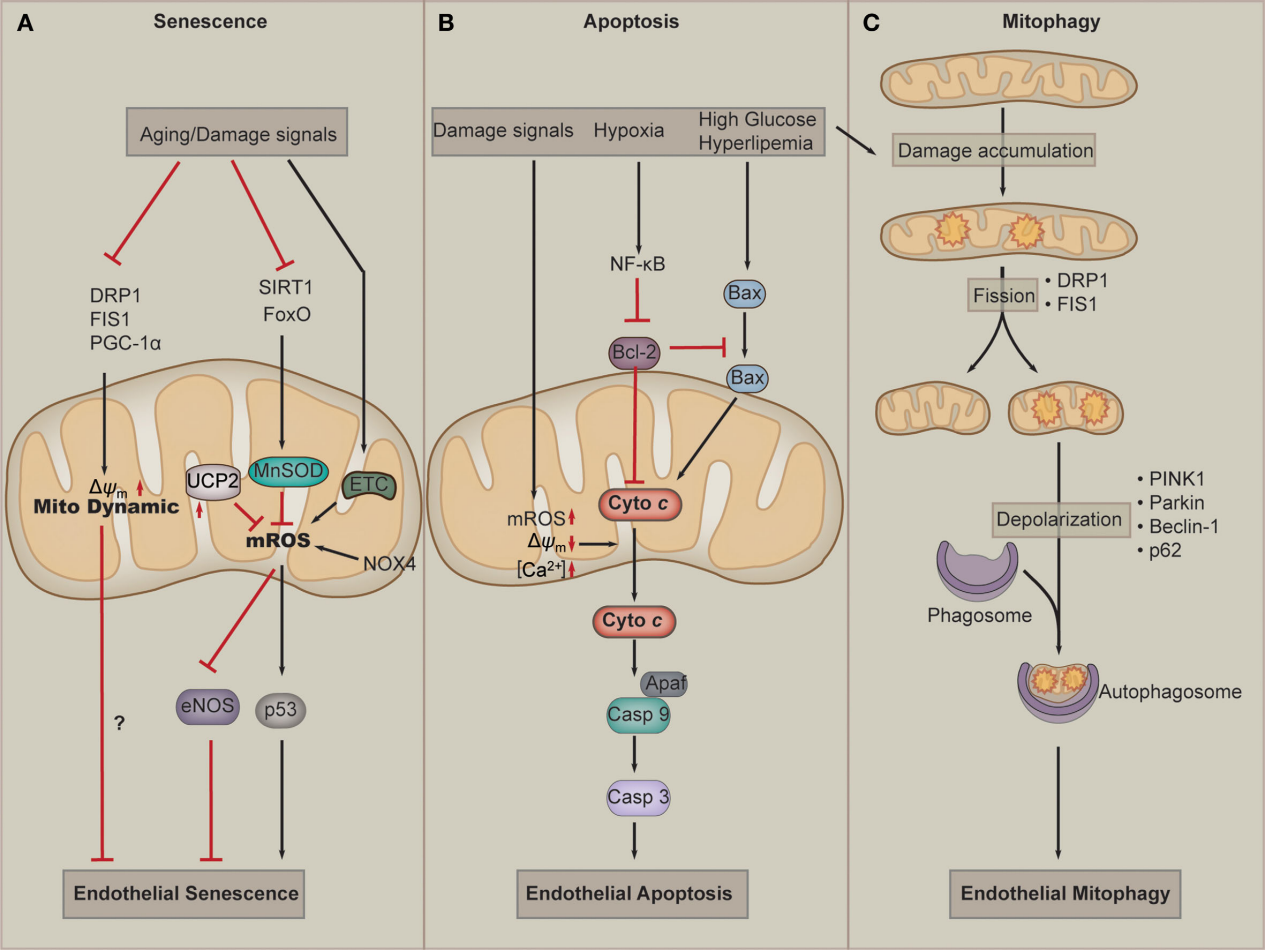
**Mitochondria in endothelial cell senescence, apoptosis, and mitophagy**. **(A)** Aging and other damage signals such as hyperglycemia inhibit the activation of dynamic proteins, such as dynamin-related protein-1(Drp1), fission 1(Fis1), and PGC-1α, which leads to the defects of mitochondria dynamics and endothelial senescence. When exposed to senescent signals, cellular SIRT1 and FoxO are downregulated, which results in downregulation of the antioxidant MnSOD. At the same time, the defected ETC and NOX4 produce more mROS. Those facts leads to accumulation of mROS, which in turn results in eNOS uncoupling and p53 activation, and subsequently endothelial cell senescence. **(B)** Damage signals lead to a loss of transmembrane potential (Δψ_m_), accumulation of mROS and Ca^2+^. These effects of mitochondrial dysfunction contribute to the release of cytochrome *c*, which activates Caspase 9-Apaf complex and leads to apoptosis by activating Caspase 3. Hyperglycemia or hyperlipemia can promotes Bax to move to the membrane of mitochondria (which can be inhibited by Bcl-2) to promote the release of cytochrome *c*. **(C)** During normal lifespan of mitochondria and in the settings of increased oxidative stress, damage to mitochondrial components accumulates. Fission, which is mediated by Drp1 and Fis1, provides a mechanism to isolate damaged components for elimination. The damaged mitochondria undergo mitophagy. Mitophagy involves mitochondrial depolarization, retention phosphatase and tensin homolog-induced putative kinase protein 1(PINK1) in the mitochondrial membrane, and recruitment of Parkin, which targets the mitochondria to autophagosome. Beclin1 and p62 also play a role in targeting cargo to the autophagosome and are subsequently degraded during active autophagy.

Over the last decade, accumulating evidence has suggested a causative link between mitochondrial dysfunction and major phenotypes associated with endothelial senescence (Figure 3A). EC senescence is associated with impaired mitochondrial biogenesis, reduced mitochondrial mass and altered expression of components of the ETC and other mitochondrial components (Ungvari et al., 2008; Dai et al., 2012). Somatic mtDNA mutations and respiratory chain dysfunction accompany normal endothelial senescence. Mitochondrial superoxide production increases with replicative senescence. Damaged mitochondria produce excessive superoxide and H_2_O_2_, which are major determinants of telomere-dependent senescence at the single-cell level that is responsible for cell-to-cell variation in replicative lifespan (Passos et al., 2007). Dysfunction of the ETC critically participates in endothelial senescence. Deficiency of mitochondrial ETC complex IV plays an essential role in senescence-induced mitochondrial dysfunction. In senescent pulmonary artery ECs, the catalytic activity of complex IV decreases by 84%, and the protein level of this subunit is also reduced in senescent ECs. The downregulation of complex IV is mediated by reduced synthesis and enhanced degradation of the mRNA (Zhang et al., 2002). In addition, in senescent ECs, the mitochondrial antioxidant MnSOD, which is regulated by FoxO and SIRT1, is significantly downregulated, resulting in damaged capacity of mROS buffering of mitochondria (Minamino and Komuro, 2007; Zhou et al., 2011b). In the absence of UCP2, endothelial growth stimulation provokes mitochondrial network fragmentation and premature senescence *via* a mechanism involving superoxide-mediated p53 activation (Shimasaki et al., 2013). In addition to mROS, the change in mitochondrial morphology, such as interconnected mitochondria, is observed in senescent ECs (Jendrach et al., 2005). Mitochondria of senescent HUVECs show a significant and equal decrease in both fusion and fission activity, indicating that these processes are sensitive to aging and could contribute to the accumulation of damaged mitochondria during aging (Jendrach et al., 2005). Decreased expression of Drp1 and Fis1, two proteins regulating mitochondrial fission, mediates mitochondrial elongation in senescent cells (Mai et al., 2010). Nox4 serves as an important orchestrator of ECs senescence. Nox4 appears to maintain an highly interconnected mitochondrial network, which may influence mitochondrial fission and/or fusion mechanisms in a manner that could be a contributing factor in the loss of replicative lifespan seen in senescence (Koziel et al., 2013). Altered mitochondrial quality control has been shown to correlate to endothelial dysfunction in aging. One of such examples is that disordered mitochondrial dynamics and loss of Δψ_m_ are observed in cell culture models of senescence in ECs. Improved mitochondrial fitness, as evidenced by higher Δψ_m_, increased ATP production, and decreased damage to mtDNA, is associated with prolonged lifespan of cultured ECs (Mai et al., 2012). PGC-1α is the central regulator for mitochondrial biogenesis and dynamics. A recent work has identified PGC-1α as a negative regulator of vascular senescence (Xiong et al., 2013). Altogether, current evidence implies that dysfunction of mitochondrial ROS buffering activity and mitochondrial dynamics play a key role in EC senescence.

Mitochondria are implicated in cell death pathways, including apoptosis and necrosis, which has been reviewed elsewhere (Wang and Youle, 2009; Tait and Green, 2010). Mitochondria are central mediators of apoptosis in ECs (Figure 3B). Intrinsic apoptosis is initiated by cellular stressors, including hypoxia, ROS, oxidized low density lipoproteins (ox-LDL), and DNA damage. Such stimuli activates BH3-only proteins, which inhibit antiapoptotic factors, including B-cell lymphoma 2 (Bcl-2) and allow activation of Bcl-2-associated X protein (BAX) (Kluge et al., 2013). Supplementation of ECs with mitochondria-targeted antioxidants inhibits peroxide-induced mitochondrial iron uptake, oxidative damage, and apoptosis (Dhanasekaran et al., 2004). Ox-LDL induces dysfunction of the Δψ_m_, leading to cytochrome *c* release into the cytosol, and thereby stimulates apoptosis of human ECs. Apoptosis suppression by CSA correlates with the prevention of mitochondrial dysfunction and thus indicates the importance of mitochondrial destabilization in ox-LDL-induced apoptosis (Walter et al., 1998). Continuous oxidation of high density lipoprotein (HDL) under hyperglycemic conditions may induce endothelial apoptosis through a mitochondrial dysfunction, following the deterioration of vascular function (Matsunaga et al., 2001). High glucose increases intracellular ROS and cell apoptosis through a mechanism involving interregulation between cytosolic and mROS generation. C-peptide activation of AMP protein kinase α subunit (AMPKα) inhibits high glucose–induced ROS generation, mitochondrial fission, Δψ_m_ collapse, and EC apoptosis (Bhatt et al., 2013). PGC-1α regulates ROS generation and apoptosis in ECs by increasing fatty acid oxidation and enhancing ATP/ADP translocate activity (Won et al., 2010). FOXO3a governs early and late apoptotic endothelial programs during elevated glucose through mitochondrial and caspase signaling (Hou et al., 2010). Factors regulating the release of cytochrome *c* critically participate in mitochondria-dependent apoptosis of EC. A1, one of Bcl-2 family members, is localized to and functions in mitochondria. A1 is able to repress mitochondrial depolarization, loss of cytochrome *c*, cleavage of caspase 9, BID and poly(ADP-ribose) polymerase. A1 maintains temporary survival of ECs in response to TNF-α by maintaining mitochondrial viability and function (Duriez et al., 2000). Apoptosis signal-regulating kinase 1 (ASK1) mediates cytokines and ROS-induced apoptosis in a mitochondria-dependent pathway. Overexpression of thioredoxin-2 inhibits ASK1-induced apoptosis without effects on ASK1-induced JNK activation in EC. Moreover, specific knockdown of thioredoxin-2 in EC increases TNF/ASK1-induced cytochrome *c* release and cell death without increase in JNK activation, Bid cleavage, and Bax translocation (Zhang et al., 2004). Hepatocyte growth factor (HGF), which is a novel member of the angiogenic growth factors, can also inhibits cytochrome *c* release and EC apoptosis by upregulating the level of Bcl-2 (Nakagami et al., 2002). In addition to apoptosis, mitochondria are also involved in necrosis. Mitochondria-mediated necrosis critically participates in cardiac myocyte dysfunction and cell death. However, whether mitochondria function in EC necrosis remains elusive. A recent report has showed that mitochondria do not contribute to TNF-α-induced necrosis in SVEC cells, an established murine endothelial cell line (Tait et al., 2013). However, it remains to explore whether mitochondria participate in necrosis in ECs from other tissues or species, or whether pathological stimuli can induce EC necrosis.

Autophagy is an evolutionally conserved cellular process in which cells “eat” themselves to fill the energy demand or to recycle substrates or organelles that are damaged. Mitophagy refers to the selective autophagy of mitochondria. As mitochondrial damage accumulates, networks undergo rearrangement and fission to yield different populations of daughter mitochondria. While the normal daughter mitochondria re-enter the mitochondria life cycle, the damaged ones undergo mitophagy to reduce damage such as mROS (Figure 3C). Accumulating evidence suggests that impaired mitophagy contribute to the pathogenesis of vascular diseases including diabetes mellitus, atherosclerosis, and hypertensive heart diseases. Several recent studies have examined the involvement of mitophagy in ECs under conditions of oxidative stress and energy deprivation. Oxidative damage induced by mitochondria-targeted irradiation of ECs promotes Parkin translocation to depolarized mitochondria and increases LC3-II level and autophagosome formation (Mai et al., 2012). When exposed to hemin, ECs undergo lipid peroxidation, leading to mitochondria depolarization and mitophagy (Higdon et al., 2012). However, it is also reported that beclin-1/LC3-II-mediated autophagy is also a mechanism for ECs to cleat ox-LDL as well. Taken together, autophagy and mitophagy are important responses of ECs to oxidative stress.

In summary, mitochondria critically participate in endothelial cell senescence, apoptosis and mitophagy, and the three aspects are important for endothelial cell function. However, those three processes are individually known in EC. How they crosstalk to regulate endothelial function remains unknown. It is interesting to explore whether mitochondria could cooperate those processes in EC.

## Endothelial mitochondria in vascular diseases

**Figure 4:**
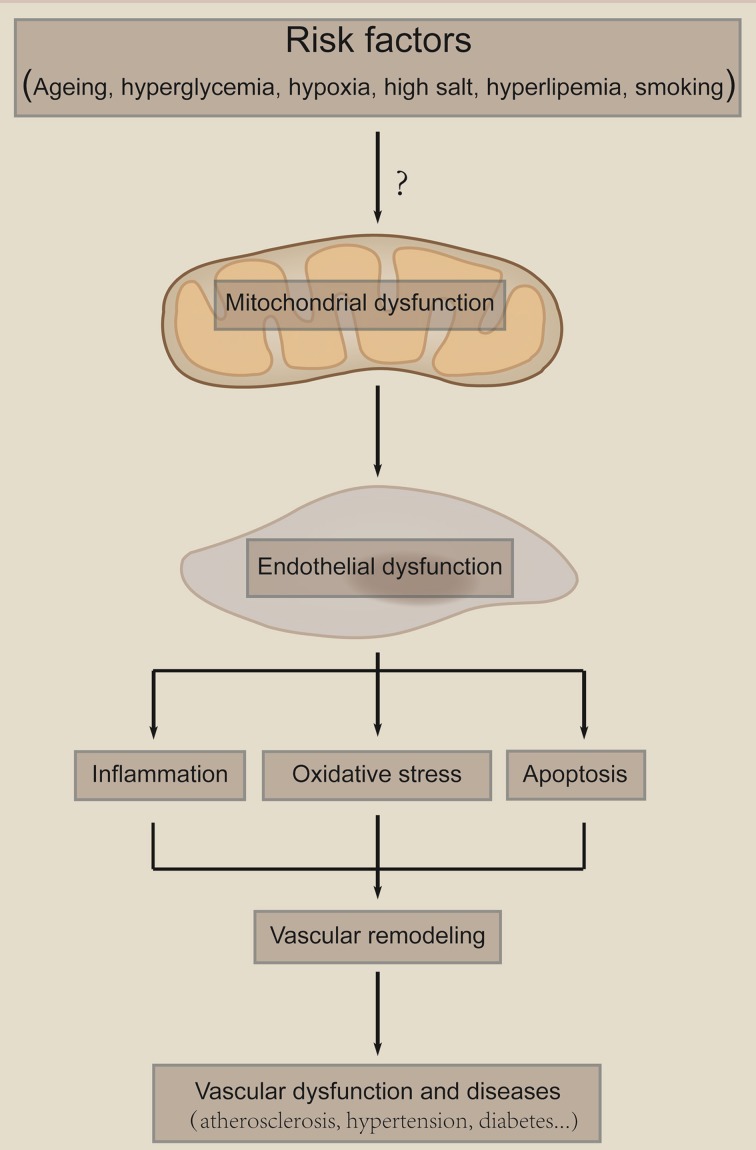
**Mitochondria as a sensor of damage signals**. Mitochondria serve as a sensor of environmental damage signals, such as hyperglycemia, aging, hypoxia, high salt, and smoking. Those risk factors cause mitochondria damage and subsequent endothelial dysfunction. Endothelial dysfunction leads to inflammation, oxidative stress and cell death, which result in vascular remodeling and subsequent vascular diseases. The “?” means that it is largely unknown how risk factors induce mitochondria dysfunction.

Endothelial dysfunction has been linked to a variety of disease states, including atherosclerosis, diabetes mellitus, coronary artery disease, hypertension, and hypercholesterolemia. Mitochondria-mediated dysfunction of ECs are critical in those diseases. Endothelial mitochondria serve as a pivotal sensor of the local environment and transduce damage signals, which leads to mitochondria damage, endothelial dysfunction, vascular remodeling and vascular diseases (Figure 4). Here we focus on atherosclerosis, diabetic endothelial dysfunction, PAH and hypertension to discuss the role of endothelial mitochondria in vascular diseases.

### Atherosclerosis

Atherosclerosis is a chronic disease of the arterial wall, which is a leading cause of death and loss of productive life years worldwide. Atherosclerosis begins with the recruitment of inflammatory cells to the intima. Therefore, endothelial layer is the first barrier against atherosclerosis, and endothelial dysfunction is frequently involved in atherosclerosis.

Atherosclerosis is associated with a number of metabolic disturbances including diabetes, abnormal lipid metabolism, obesity; and metabolism, implicating mitochondrial component. Interestingly, the endothelial mitochondria themselves participate in atherosclerosis. One of the major mechanisms underlying endothelial mitochondria participate in atherosclerosis is elevated mROS, which leads to endothelial dysfunction or apoptosis, the earliest event; and inflammation, one of the most dominant features of arthrosclerosis. What is important is that ECs are more sensitive to reactive species-mediated damage than smooth muscle cells (Ballinger et al., 2000). mROS is increased in response to many atherosclerosis inducers, including ox-LDL, triglycerides, and hyperglycemia. For example, exposure of ECs to free fatty acids, which upregulate in patients with metabolic syndrome, increases mROS (Du et al., 2006). Otherwise, hyperglycemia changes mitochondrial dynamics and increases mROS in ECs, while the normalization of blood sugar inhibits the progression of vascular damage (Nishikawa et al., 2000; Yu et al., 2006; Shenouda et al., 2011). ROS produced in the vascular microenvironment causes mitochondrial damage and dysfunction, which in turn amplifies this effect partly due to reduced ROS buffering capacity. Mitochondrial protein synthesis is inhibited in a dose-dependent manner by ONOO^−^, resulting in decreased cellular ATP levels and mitochondrial redox function (Ballinger et al., 2000). Reduced synthesis of NO contributes to the endothelial dysfunction and may be related to limited availability of L-arginine, the common substrate of constitutive NOS and cytosolic arginase I and mitochondrial arginase II. Mitochondrial arginase II modulates NO synthesis through nonfreely exchangeable L-arginine pools in human ECs. Selective endothelial overexpression of arginase II induces endothelial dysfunction and enhances atherosclerosis in mice (Vaisman et al., 2012). Atherosclerosis actually happen in old people and mROS is elevated in aged vascular tissues. Vascular ECs with senescence-associated phenotypes are present in human atherosclerotic lesions, and EC senescence induced by telomere shortening may contribute to atherosclerosis (Minamino et al., 2002).

Another vulnerable target of mROS is mtDNA, owing to ETC proximity and the relative lack of mtDNA repair mechanisms. Elevated mtDNA damage indwells in human atherosclerotic samples compared with age-matched transplant donors. In atherosclerosis-prone ApoE^−/−^ mice and in human arterial specimens, the extent of atherosclerosis correlates with mtDNA damage (Ballinger et al., 2002). Since mitochondrial mutations may lead to production of more ROS, it may initiate a cycle of positive feedback. Failure of DNA repair generates defects in cell proliferation, apoptosis, and mitochondrial dysfunction, which in turn leads to ketosis, hyperlipidemia, and increased fat storage, promoting atherosclerosis and metabolic syndrome. In addition, this may also implicate the possibility that inherited mtDNA damage mutations could even initiate vascular damage and increase the risk of atherosclerosis (Nomiyama et al., 2004; Abu-Amero and Bosley, 2006; Sobenin et al., 2012a,b, 2013). A recent report has shown that mtDNA damage can promote atherosclerosis independently of ROS through effects on smooth muscle cells and monocytes and correlates with higher risk plaques in human (Yu et al., 2013). However, the relationship between atherosclerosis and mitochondrial mutations in ECs remains elusive.

Factors that modulate endothelial mROS production are evidenced to participate in atherosclerosis. One of such examples is the adaptor protein, p66^Shc^. The fact that the expression of p66^Shc^ may be relevant in cardiovascular function is evident from the observations that p66^Shc^ knockout mice are protected against ROS-dependent, age-related endothelial dysfunction (Francia et al., 2004), hyperglycemia-induced endothelial dysfunction (Zhou et al., 2011b), as well as high-fat-induced atherosclerosis (Napoli et al., 2003; Martin-Padura et al., 2008). The function of p66^Shc^ is regulated by SIRT1 at the chromatin level (Zhou et al., 2011b). Previous work in our laboratory showed that endothelial-specific SIRT1 transgenic mice exhibit smaller atherosclerotic lesions, which may be partly attributed to the reduced local ROS in the lesions (Zhang et al., 2008). This effect may be at least partly contributed by the effects of SIRT1 on endothelial mitochondria, because resveratrol, a nature activator of SIRT1, can attenuates mitochondrial oxidative stress in coronary arterial ECs (Ungvari et al., 2009). In addition, endothelial-specific expression of mitochondrial thioredoxin-2 improves endothelial cell function and reduces atherosclerotic lesions. Thioredoxin-2 TG mice have increased total antioxidants, reduced oxidative stress, and increased NO levels in serum compared with their control littermates. Consistently, aortas from thioredoxin-2 TG mice show reduced vasoconstriction and enhanced vasodilation (Zhang et al., 2007).

Current research focuses mainly on the oxidation-reduction process mediated by mitochondria. Other roles of mitochondria in mediating endothelial function in atherosclerosis remain elusive. Our current understanding indicates that atherosclerosis is a metabolic disease and endothelial cell mentalism is important for their function in the artery. However, it is unknown whether mitochondria-mediated alteration of metabolism in EC participates directly in atherosclerosis.

### Diabetic vascular dysfunction

Diabetes mellitus is associated with an increased risk of cardiovascular disease even in the presence of intensive glycemic control. Vascular ECs are an important target of hyperglycaemic damage. Mitochondrial dysfunction plays a central role in endothelial dysfunction in type II diabetes mellitus (Kizhakekuttu et al., 2012). In type II diabetes patients, mitochondrial function is impaired, which is evident from lower mitochondrial O_2_ consumption, Δψ_m_, polymorphonuclear cell rolling velocity, and GSH/GSSG ratio, and higher mROS production and rolling flux (Hernandez-Mijares et al., 2013).

Hyperglycemia-induced increase in the production of ROS by the mitochondrial ETC in EC has been implicated in glucose-mediated vascular damage (Nishikawa et al., 2000; Brownlee, 2001; Du et al., 2003). Activation of AMPK reduces hyperglycemia-induced mROS production and promotes mitochondrial biogenesis in HUVECs (Kukidome et al., 2006). In both mature ECs and EPCs, AMPK activation by its agonists suppresses high-glucose-induced ROS generation by promoting mitochondrial biogenesis (Kukidome et al., 2006), inhibiting NADPH oxidase activity (Ceolotto et al., 2007), and inducing the expression of both mitochondrial UCP2 (Xie et al., 2008), and MnSOD (Wang et al., 2011b). Endothelium-selective activation of AMPK prevents diabetes mellitus-induced impairment in vascular function and reendothelialization (Li et al., 2012). In this regard, AMPK activator such as metformin could serve as candidate drug to improve mitochondria and subsequent endothelial function. Interestingly, a recent report has showed that mROS enhances AMPK activation in the endothelium of patients with coronary artery disease and diabetes (Mackenzie et al., 2013). This finding may implicate that high glucose induces endothelial dysfunction by upregulating mROS, which in turn leads to the activation of AMPK. This feedback pathway may be a conserved pattern for the body to protect itself. Hyperglycemia inhibits thioredoxin ROS-scavenging function through induction of thioredoxin-interacting protein (Txnip), which interacts with thioredoxin and serves as an endogenous inhibitor (Schulze et al., 2004; Li et al., 2009). Overexpression of Txnip increases oxidative stress, while Txnip gene silencing restores thioredoxin activity in hyperglycemia (Schulze et al., 2004). In addition, Txnip induces inflammation through chromatin modification in retinal capillary EC under diabetic conditions (Perrone et al., 2009). Importantly, diabetic animals exhibit increased vascular expression of Txnip and reduced thioredoxin activity, which normalizes with insulin treatment (Schulze et al., 2004).

Mitochondria also contribute to hyperglycemia-induced EC apoptosis. In addition to mROS overproduction, other pathways have essential roles in this process. Mitochondria depolarization has been implicated in hyperglycemia-induced apoptosis of human aortic ECs. Apoptosis in human aortic ECs induced by hyperglycemia involves mitochondrial depolarization and mROS overproduction, which is prevented by the antioxidant N-acetyl-L-cysteine (Recchioni et al., 2002). ROCK1 is a potent regulator of mitochondrial dynamics in diabetic nephropathy and Drp1 is a direct substrate for ROCK1. In hyperglycemic conditions, ROCK1 phosphorylates Drp1 and leads to mitochondrial fission, mROS production and subsequent release of cytochrome *c* (Wang et al., 2012). In retinal ECs, high glucose downregulates mitochondrial connexin 43, which leads to mitochondria shape change and cytochrome *c* release (Trudeau et al., 2012). Hyperglycemia-induced mitochondrial fragmentation with concomitant increase in Δψ_m_ heterogeneity, reduced oxygen consumption, and cytochrome *c* release may underlie apoptosis of retinal EC as seen in diabetic retinopathy (Trudeau et al., 2010). The mitochondrial permeability transition pore (mPTP) is an oxidative stress–sensitive channel involved in cell death. Elevated glucose concentration leads to an oxidative stress that favors mPTP opening and subsequent cell death in several endothelial cell types and metformin prevents this mPTP opening–related cell death (Detaille et al., 2005).

For a long time, it was unknown why vascular damage still occurs in diabetes patients even in the presence of intensive glycemic control. Hyperglycemic memory may explain why intensive glucose control has failed to improve cardiovascular outcomes in patients with diabetes. Indeed, hyperglycemia promotes vascular dysfunction even after glucose normalization. Accumulating observations support the concept that ROS-driven hyperglycemic stress is remembered in the vasculature. The mitochondrial adaptor protein p66^Shc^ critically participates in the hyperglycemic memory in vascular ECs. In human aortic ECs exposed to high glucose and aortas of diabetic mice, activation of p66^Shc^ by protein kinase C β II (PKCβII) persist after returning to normoglycemia. Persistent p66^Shc^ upregulation and mitochondrial translocation are associated with continued ROS production, reduced NO bioavailability, and apoptosis. *In vitro* and *in vivo* gene silencing of p66^Shc^, performed at the time of glucose normalization, blunts ROS production, restores endothelium-dependent vasorelaxation, and attenuates apoptosis by limiting cytochrome *c* release, caspase 3 activity, and cleavage of PARP (Paneni et al., 2012). Our previous study showed that SIRT1 inhibited high-glucose-induced p66^Shc^ upregulation in HUVECs. Moreover, compared with streptozotocin-induced wild-type diabetic mice, endothelium-specific SIRT1 transgenic diabetic mice had decreased p66^Shc^ expression, improved endothelial function, and reduced accumulation of nitrotyrosine and 8-OHdG (Zhou et al., 2011b). This finding implicates that SIRT1 may be an important regulator of hyperglycemic memory. The relationship among SIRT1, p66^Shc^, oxidative stress, damage memory and endothelial senescence has been discussed previously (Chen et al., 2013).

In conclusion, mitochondria are essential for hyperglycemia-induced endothelial dysfunction. Mitochondria function in this process through at least three pathways: mROS production, apoptosis and damage memory. Hyperglycemia upregulates the production of mROS and inhibits activity of the endothelial ROS buffering system, which leads to damage of mtDNA and other mitochondrial components that are important for normal endothelial function. In addition, the balance between antiapoptotic and proapoptotic pathways is broken. Therefore, current research has deeply investigated the participation of mitochondria in hyperglycemia-induced endothelial dysfunction. However, how mitochondria-mediated endothelial dysfunction contributes to secondary vascular diseases, such as atherosclerosis, remains unclear.

### Pulmonary artery hypertension

PAH is an ideal vascular disease to discuss for the reason that it reflects all the function of mitochondria so far. PAH is caused by excessive proliferation of vascular cells such as smooth muscle cells and ECs that eventually obliterate the pulmonary arterial lumen, and lead to right ventricular failure and premature death. The cause of the vascular remodeling in PAH remains elusive and the prognosis of PAH is still poor. Abnormal mitochondria in PAH pulmonary arteries suppress mitochondria-dependent apoptosis and contribute to vascular remodeling. Although many investigations in this field focus on smooth muscle cells, the role of EC attracts increasing attentions. Similar to atherosclerosis, EC dysfunction and apoptosis appears to be an early event in PAH. However, later stages are characterized by the presence of hyperproliferative and apoptosis-resistant ECs and smooth muscle cells. These cells exhibit a metabolic profile strikingly similar to that of cancer cells (Dromparis and Michelakis, 2013).

In PAH, mitochondrial signaling regulates both the acute and the chronic response of the pulmonary circulation to hypoxia, and defect in mitochondrial glucose oxidation contributes to the apoptosis-resistance and proliferative diathesis. The switch provides several advantages to EC, including: (i) diversion of pyruvate into anabolic pathways; (ii) suppression of apoptosis by hyperpolarized Δψ_m_; (iii) inhibition of Kv channels due to decreased mROS, increasing cytosolic Ca^2+^, which in turn activates hyperproliferative transcription factor nuclear factor of activated T cells (NFAT), whose activation causes downregulation of Kv channels and upregulation of glycolytic enzymes; and (iv) activation of the HIF1, which increases pyruvate dehydrogenase kinase (PDK) expression, thus sustaining mitochondrial suppression in another reinforcing feedback loop (Dromparis and Michelakis, 2013). For instance, lactate dehydrogenase A converts pyruvate to lactate necessary to sustain rapid flux through glycolysis. Pulmonary microvascular endothelial cells (PMVEC) utilize aerobic glycolysis to sustain their rapid growth rates, which is dependent on lactate dehydrogenase A (Parra-Bonilla et al., 2010).

The primary role of mitochondria in vascular ECs may be not to produce ATP but, under the control of NO, to act as signaling organelles using either oxygen of oxygen-derived species as signaling molecules. At a low oxygen concentration, endogenous NO plays a key role in preventing the accumulation and stability of HIF1α. At higher oxygen concentrations, NO facilitates the production of mROS (Quintero et al., 2006). Oxygen consumption of PAH cells is decreased, especially in state 3 respiration with substrates glutamate-malate or succinate, and this decrease parallels reduction in complex IV activity and PAH cellular NO synthesis. PAH pulmonary artery ECs have decreased mitochondrial dehydrogenase activity and lowered mitochondrial numbers per cell and mtDNA content, all of which increase after exposure to NO donors (Xu et al., 2007). Alterations of NO and MnSOD contribute to pathological HIF-1α expression and account for lower numbers of mitochondria in PAH-EC (Fijalkowska et al., 2010). Asymmetric dimethylarginine (ADMA) is an endogenous competitive inhibitor of NOS. Elevated ADMA levels are observed in numbers of conditions affecting the cardiovascular system. Recently, ADMA is shown to increase in Shunt lambs secondary to a decrease in dimethylarginine hydrolases (DDAH) activity and that ADMA increases the nitration of mitochondrial proteins in cultured lamb pulmonary arterial endothelial cells (PAEC) (Sud et al., 2008; Sun et al., 2011). Treatment of Shunt lambs with L-arginine prevents the ADMA-mediated mitochondrial redistribution of eNOS, the nitration-mediated inhibition of CrAT, and maintains carnitine homeostasis. In return, ATP levels and eNOS/heat shock protein 90 interactions are preserved, which decreases NOS uncoupling and enhances NO generation (Sun et al., 2013b).

Taken together, endothelial mitochondria participate in PAH through two main ways: aerobic glycolysis to provide substrates for cell growth and to improve the ROS system to promote cell proliferation and inhibit cell apoptosis. However, current studies focus mainly on the role of smooth muscle cells in PAH; further experimental investigations are needed to estimate the role of mitochondrial regulation of endothelial dysfunction in this disease.

### Hypertension

Hypertension is a condition associated with oxidative stress, endothelial dysfunction, and increased vascular resistance, representing probably both a cause and a consequence of elevated levels of ROS and nitrogen species. Mitochondrial dysfunction, preceding endothelial dysfunction, might favor the development of hypertension. Genetic studies have implicated the role of mitochondrial in hypertension. Gly482Ser polymorphisms in PGC-1α, a factor controlling mitochondria biogenesis, are associated with blood pressure and hypertension among Austrian men and white subjects (Oberkofler et al., 2003; Cheurfa et al., 2004; Andersen et al., 2005). A study carried out in Korean population correlates age-dependent polymorphisms in the mitochondria-shaping gene, *OPA1*, with blood pressure and hypertension (Jin et al., 2011). Mitochondrial dysfunction caused by mitochondrial tRNA^lle^ 4263A>G mutation is involved in essential hypertension (Wang et al., 2011a).

In situations of metabolic perturbation, increased mROS generation might trigger EC dysfunction, possibly contributing to the development of hypertension (Puddu et al., 2008). Thioredoxin 2, a mitochondria specific antioxidant enzyme, can attenuate Ang-II-induced hypertension (Widder et al., 2009). Nox2 contributes to mROS production and EC dysfunction (Nazarewicz et al., 2013). Nox2 depletion in gp91phox knockout mice inhibits Ang-II-induced cellular and mROS and attenuates hypertension (Dikalov et al., 2014). Ang-II induces endothelial mROS production. Overexpression the mitochondrial MnSOD elevates both basal and Ang-II-stimulated cellular superoxide. Furthermore, transgenic mice overexpressing mitochondria MnSOD attenuates Ang-II induced hypertension (Dikalova et al., 2010).

Compared with the studies on atherosclerosis, diabetes and PAH, much less work has been carried out in hypertension, and those studies mainly focus on the mROS. It is interesting to investigate other roles of mitochondria in hypertension. For instance, eNOS is important for endothelial cell function and eNOS uncoupling is observed in hypertension of animal models. Whether mitochondria participate in this process remains unknown.

## Mitochondria targeting intervention

Risk factors induce mitochondrial dysfunction in the EC, which contributes to the pathogenesis of various vascular diseases. Those findings prompt the speculation that interventions that restore mitochondrial function or “re-educate” mitochondria may be protective in endothelial dysfunction and related vascular diseases. Here we discuss mitochondria-directed antioxidants and interventions that improve mitochondria functions.

### Mitochondria-directed antioxidants

Mitochondria-derived ROS are important for signaling and EC dysfunction in vascular system, a strategy reguiding ROS to physiological levels likely will be effective. Although clinical trials of non-targeted antioxidants (such as vitamins A or E, selenium, or β-carotene) have not shown to function well, mitochondria-targeting antioxidants may offer improved efficacy. Because mitochondria are the most negatively charged organelles. Positively charged molecules have a preferential up-take in mitochondria and achieve mitochondrial concentrations as much as 1000-fold higher than in the cytoplasm. Therefore, one strategy to target mROS is to link antioxidant compounds with a lipophilic cation, such as triphenylphosphonium (TPP) (Dai et al., 2012; Smith et al., 2012). Several such agents have been designed.

Mitoquinone (mitoQ) is reduced to ubiquinol within the mitochondrial matrix. Investigators enhance mitochondrial function selectively by attaching mitoQ to TPP *via* a long lipophilic alkyl chain (James et al., 2005). Administration of the mitochondria-targeted antioxidant mitoQ protects against the development of hypertension, improves endothelial function, and reduces cardiac hypertrophy in young stroke-prone spontaneously hypertensive rats (Graham et al., 2009). Furthermore, mitoQ has been shown to prevent diabetic nephropathy and cardiac dysfunction (Chacko et al., 2010; Vergeade et al., 2010). MitoQ has been used to target mROS in many nonvascular diseases in animals and even in early-phase clinical trials (Smith and Murphy, 2010). A recent work demonstrated that mitoQ treatment reduced the macrophage content and cell proliferation within plaques of atherosclerosis (Mercer et al., 2012).

A similar strategy has been performed to deliver α-tocopherol or the mitochondria-targeting TEMPOL (mitoTEMPO) to mitochondria. Treatment with mitoTEMPO attenuates hypertension when given at the onset of Ang-II infusion and decreases blood pressure by 30 mm Hg following establishment of both Ang-II-induced and DOCA salt hypertension, whereas a similar dose of non-targeted TEMPO was not effective. *In vivo*, mitoTEMPO decreases vascular O^▪−^_2_, increases vascular NO production and improves endothelial-dependent relaxation. Interestingly, transgenic mice overexpressing MnSOD show attenuated Ang-II-induced hypertension and vascular oxidative stress similar to mice treated with mitoTEMPO (Dikalova et al., 2010). Another mimetics of MnSOD is metalloporphyrin Mn (III) tetrakis (4-benzoic acid) porphyrin (MnTBAP). Administration of MnTBAP reverses the hyperproliferative PAH phenotype *in vitro* and *in vivo* (Archer et al., 2010).

Some antioxidants, which may be not designed to target mitochondria, are shown to improve mitochondria function significantly. One of such examples is the vitamin D, which has been known to be important in many cellular functions of several tissues and organs other than bone. Vitamin D receptors have been found in all the major cardiovascular cell types including cardiomyocytes, arterial wall cells, and immune cells (Norman and Powell, 2014). Vitamin D alone or in combination with ZK191784 is able to prevent the loss of mitochondrial potential and the consequent cytochrome *c* release and caspase activation in HUVEC undergoing oxidative stress (Uberti et al., 2013). Moreover, vitamin D is a regulator of eNOS and arterial stiffness in mice (Andrukhova et al., 2013). Vitamin D insufficiency is associated with depletion of circulating endothelial progenitor cells and endothelial dysfunction in patients with type 2 diabetes (Yiu et al., 2011). A single large dose of oral vitamin D improves endothelial function in patients with type 2 diabetes (Sugden et al., 2008). Nevertheless, the role of vitamin D supplementation in the management of cardiovascular disease remains to be established.

### Caloric restriction and mimetics

Caloric restriction (CR) is a dietary regimen that offers benefits by improving mitochondria function and quantity control. CR decreases mROS at complex I and lowers oxidative damage to mtDNA in the rat heart (Gredilla et al., 2001). In animal models and human patients, CR increases mitochondrial biogenesis and bioenergetic efficiency (Nisoli et al., 2005; López-Lluch et al., 2006; Civitarese et al., 2007). CR was first reported to lower blood pressure in the spontaneously hypertensive rat 35 years ago (Young et al., 1978). A recent work has showed that CR ameliorates Ang-II-induced cardiomyocytes hypertrophy, vascular inflammation partly through reprogramming mitochondria proteomic profile in rats (Finckenberg et al., 2012). In addition, CR reduces atherosclerosis and oxidative stress in the aorta of ApoE^−/−^ mice (Guo et al., 2002). Importantly, CR can significantly reduce the onset of cardiovascular diseases in monkeys (Colman et al., 2009). In human, long-term CR is highly effective in reducing the risk for atherosclerosis and hypertension (Fontana et al., 2004). CR alone and with exercise reduces CVD risk in healthy non-obese individuals (Lefevre et al., 2009). CR is shown to regulate several pivotal orchestrators in metabolic, including AMPK, SIRT1, and mammal target of rapamycin (mTOR) as well as insulin-like growth factors (Fontana et al., 2010). Activators/inhibitors of those proteins are demonstrated to be CR mimics and mediate mitochondria function.

Resveratrol is an activator of AMPK and SIRT1. Treatment of rats with resveratrol increases expression of eNOS, decreases oxidative stress, and improves endothelial function in small pulmonary arteries. Resveratrol prevents monocrotaline-induced PAH in rats (Csiszar et al., 2009). In addition, resveratrol suppresses atherosclerosis in hypercholesterolemic rabbits without affecting plasma lipid levels (Wang et al., 2005). Metformin, which activates AMPK, has been shown to inhibit mPTP opening and endothelial cell apoptosis and to prevent endothelial dysfunction in experimental models (Schulz et al., 2008) and to stimulate microvascular repair in acute lung injury (Jian et al., 2013). The thiazolidinediones, including pioglitazone, have been reported to activate PGC1-α, a downstream factor of AMPK and SIRT1, and enhance mitochondrial biogenesis in ECs (Fujisawa et al., 2009). Rapamycin, the inhibitor of mTOR, is evidenced to attenuate atherosclerosis (Waksman et al., 2003), hypertension and PAH (Morales et al., 2001; Nishimura et al., 2001).

Taken together, accumulating evidence demonstrates that mitochondria-targeting antioxidants, CR and its mimetics can reduce vascular diseases. However, what we should notice here is that although those interventions can improve the function of EC, further evidence will be requested to verify the essential roles of endothelial mitochondria in those processes, although genetic approaches with endothelial specific transgene or knockout of mitochondria genes have provided strong evidence that endothelial mitochondria is involved in vascular diseases. Therefore, endothelial mitochondria may act as a promising therapeutic target to improve endothelial function and to prevent against vascular diseases.

## Concluding remarks

Mitochondria content in EC is relatively low in comparison with those with high-energy demand. EC obtain a large proportion of energy from the anaerobic glycolytic metabolism of glucose. Those facts implicate the mitochondria in EC are unlikely to act as an energy factory but, sense the local environment the EC face and orchestrate the cellular hemostasis and function. Persistent environmental risk signals can damage mitochondria, which in turn produce excessive ROS and accelerate e EC senescence, death and dysfunction. EC serve as the first barrier of the vascular system, the dysfunction of endothelial cell is considered to be the pathological basis of various vascular diseases including atherosclerosis, diabetic vascular dysfunction, PAH and hypertension. Rescuing mitochondrial function, or “re-educating” the damaged mitochondria, has been demonstrated as potential interventions to improve vascular conditions both in animal models and in human patients.

However, several interesting issues are up in the air in this field. The first issue is mitochondria-nucleus communication. Accumulating evidence have implicated that mitochondria can send signals to the nucleus, regulating the events in the nucleus. More importantly, it is interesting to see whether mitochondria signal influences epigenetic remarks in the nucleus. Acetylation and methylation of histone tails are dynamics processes regulated by histone de/acetyltransferases, methyltransferases, or demethylases. Co-factors, including flavin adenine dinucleotide (FAD), acetyl-CoA, and α ketoglutarate (α-KG), are associated with the processes of active de/methylation or de/acetylation (Minocherhomji et al., 2012). Both FAD and α-KG are known to be synthesized in mammalian mitochondria. In this regard, mitochondria are critically important for epigenetic modification in the nucleus. Altered levels of these co-factors due to mitochondrial impairment/dysfunction could have significant effects on regulation of the nuclear genome, and subsequent endothelial function. In addition, depletion of mtDNA results in significant changes in methylation pattern of a number of genes (Smiraglia et al., 2008). Vascular EC undergo senescence, apoptosis and mitophagy in disease conditions. All those processes are regulated at least in part by mitochondria. Therefore, the second question is whether mitochondria serve as pivotal modulators of those processes just as orchestrating a shadow play. Finally, with regard to mitochondria-targeted approaches, current studies are focusing on the antioxidants. It remains largely unknown the roles of dysregulated metabolites of the mitochondria in mitochondria damage and endothelial cell dysfunction. If they are important, they may serve as potential targets to “re-educate” mitochondria in EC, and subsequently serve as candidate targets for vascular diseases therapy.

### Conflict of interest statement

The authors declare that the research was conducted in the absence of any commercial or financial relationships that could be construed as a potential conflict of interest.

## Abbreviations

EC: endothelial cell
ROS: reactive oxygen species
ATP: adenosine 5'-triphosphate
ER: endoplasmic reticulum
mtDNA: mitochondrial DNA
PGC-1α: peroxisome proliferation-activated receptor γ co-activator 1α
NRF: nuclear respiratory factor
TFAM: transcription factor A mitochondrial
TFBM: transcription factor B mitochondrial
Ang-II: angiotensin II
ETC: electron transport chain
mROS: mitochondrial ROS
NADPH: nicotinamide adenine dinucleotide phosphate
NOX: NADPH oxidase
mitoK_ATP_: mitochondrial ATP-sensitive potassium channel
MnSOD: manganese superoxide dismutase
PON: paraoxonase
UCP: Uncoupling protein
Δψ_m_: mitochondrial membrane potential
HIF: hypoxia-inducible factor
NO: nitric oxide
eNOS: endothelial NO synthase
HUVEC: human umbilical vein endothelial cells
MCU: mitochondrial Ca^2+^ uniporter
MCUR1: mitochondrial calcium uniporter regulator 1
TNFR1: tumor necrosis factor receptor 1
TNF-α: tumor necrosis factor-α
mtNOS: mitochondrial NOS
PI3K: phosphoinositide-3 kinase
FoxO: Forkhead box ‘Other’
Bcl-2: B-cell lymphoma 2
BAX: Bcl-2-associated X protein
ox-LDL: oxidative low density lipoprotein
HDL: high density lipoprotein
AMPK: AMP-activated protein kinase
SVEC: SV40-transformed murine endothelial cell line
VEGF: vascular endothelial growth factor
NF-κB: nuclear factor-kappa B
PAH: pulmonary artery hypertension
FHR: Fawn hooded rat
ADMA: asymmetric dimethylarginine
DDAH: dimethylarginine hydrolases
PAEC: pulmonary arterial endothelial cells
EPC: endothelial progenitor cells
[Ca^2+^]_m_: intramitochondrial Ca^2+^
[Ca^2+^]_*c*_: cytoplasmic Ca^2+^
PARP: poly(ADP-ribose) polymerase
mPTP: mitochondrial permeability transition pore
CSE: cystathionine-γ-lyase
VCAM-1: vascular cell adhesion molecule-1
TPP: triphenylphosphonium
Mito-Q: mitoquinone
DOCA: deoxycorticosterone acetate
UV: uric acid
CR: caloric restriction.
